# TAP: A static analysis model for PHP vulnerabilities based on token and deep learning technology

**DOI:** 10.1371/journal.pone.0225196

**Published:** 2019-11-18

**Authors:** Yong Fang, Shengjun Han, Cheng Huang, Runpu Wu

**Affiliations:** 1 College of Cybersecurity, Sichuan University, Chengdu 610065, China; 2 China Information Technology Security Evaluation Center, Beijing 100085, China; Victoria University, AUSTRALIA

## Abstract

With the widespread usage of Web applications, the security issues of source code are increasing. The exposed vulnerabilities seriously endanger the interests of service providers and customers. There are some models for solving this problem. However, most of them rely on complex graphs generated from source code or regex patterns based on expert experience. In this paper, TAP, which is based on token mechanism and deep learning technology, was proposed as an analysis model to discover the vulnerabilities of PHP: Hypertext Preprocessor (PHP) Web programs conveniently and easily. Based on the token mechanism of PHP language, a custom tokenizer was designed, and it unifies tokens, supports some features of PHP and optimizes the parsing. Besides, the tokenizer also implements parameter iteration to achieve data flow analysis. On the Software Assurance Reference Dataset(SARD) and SQLI-LABS dataset, we trained the deep learning model of TAP by combining the word2vec model with Long Short-Term Memory (LSTM) network algorithm. According to the experiment on the dataset of CWE-89, TAP not only achieves the 0.9941 Area Under the Curve(AUC), which is better than other models, but also achieves the highest accuracy: 0.9787. Further, compared with RIPS, TAP shows much better in multiclass classification with 0.8319 Kappa and 0.0840 hamming distance.

## Introduction

At present, the Internet plays an important role in politics, economy, culture and social life. There are various security issues in different emerging Internet environments, such as Internet applications [1], cloud computing [2], crowdsourcing [3] and so on. With the rapid growth of open-source website applications, cyber threats are also emerging. According to an F5 Labs research on 433 major malicious attack incidents spanning 12 years, Web applications are the origin of 53% of malicious attacks [4]. Therefore, in order to resist intruders effectively, the security of the Web applications should be ensured at first.

There are 55.2% of Alexa top 10 million websites built on the content management system (CMS). WordPress, Joomla! and Drupal are the top three most popular CMSs, and their market share is 69% [5]. They are all PHP programs and the global usage of PHP programs is more extensive with the addition of other PHP Web applications. Unfortunately, some PHP programmers lack the basic knowledge of secure programming, and some vulnerabilities are difficult to detect, even for programmers with security experience.

PHP is so widely used on the Internet that any few security problems may cause a global disaster. PHPMailer is probably the most popular PHP email module in the world, which is used by many open-source projects like WordPress, Drupal, Joomla! and so on. However, there was a severe security problem—CVE-2017-5223 [6], which could allow any attackers to read servers’ local files. It affects the file content security of millions of websites servers around the world, and it would even further affect the administrative security of the servers. Another PHPMailer’s vulnerability—CVE-2016-10033 [7], could allow hackers to execute arbitrary code and threaten the security of systems directly. The National Vulnerability Database (NVD) even assigned it a 9.8/10 (CRITICAL) rating because of its great harm.

As a programming language for millions of websites, the security issue of PHP programs cannot be ignored. In the past, the security of PHP programs was assured by manual auditing, which was not only complex but also wasting time and labor. Moreover, manual auditing can not cope well with the current large open-source codes. At present, the common method is the regex pattern, and it depends on vulnerability detective features extracted from human experts’ experience. Representative tools include RIPS [8] and Pixy [9]. Those tools are much faster than traditional manual auditing, but they require features to address vulnerabilities, which is often not feasible for some complicated vulnerabilities. With the development of machine learning, more and more scholars began to seek methods in machine learning and deep learning fields. They usually manually extracted some features for traditional machine learning models or defined lots of sensitive taint functions to auxiliary detect vulnerabilities.

### Our contributions

In this paper, we proposed a static PHP source code analysis model named TAP, which bases on token and deep learning technology. TAP neither requires to extract any features vectors manually, nor defines any sensitive taint functions. In order to facilitate the processing of machine learning models, a custom tokenizer was designed. The major contributions of this paper are summarized as follows:

- In this paper, a custom tokenizer was designed based on the token mechanism of PHP. The tokenizer not only can parse source codes into tokens with more available information, but also deal with some features of PHP well.
- This paper proposed a new convenient method of performing data flow analysis. Source codes are transformed into abstract operation sequences by iterating parameter change without executing any codes.
- In this paper, TAP, which is based on the token mechanism and deep learning technology, was proposed as an analysis model to discover the PHP vulnerabilities. As far as we know, TAP is the only deep learning model that can deal with more than 7 categories of PHP vulnerabilities well. According to our experimental evaluation, TAP achieves 0.9941 AUC and 0.9787 accuracies on the CWE-89 dataset. Compared with contrasts, TAP shows much better with 0.8319 Kappa in multiclass classification.

## Related work

There are two major categories in the field of source-code static analysis. One is making tools based on expert knowledge and experience, and another is applying machine learning technology to detect vulnerabilities automatically.

There are some well-known traditional tools. Pixy [9] combines flow-sensitive, interprocedural with context-sensitive techniques for detecting vulnerabilities. But Pixy only focuses on cross-site scripting(XSS), the authors of Pixy consider that Pixy can detect other taint-style vulnerabilities like Structured Query Language(SQL) injection and command injection by some engineering effort. RIPS [8] is different from Pixy which must run in Java environment, RIPS is a PHP program that uses the inbuilt PHP tokenizer functions, and it focuses on sensitive functions and taint inputs data flow analysis. SAFERPHP [10] combines taint analysis with control-flow graph (CFG) [11] to find some semantic vulnerabilities like missing authorization problems and Denial-of-Service(DoS).

It is very inconvenient to keep on updating new detective modules for these tools. While there appear new attack skills, experts must immediately find the features of vulnerabilities and update the sensitive functions lists. For example, RIPS added some PHP magic methods in the watch list for the Property-Oriented Programming (POP) vulnerability in an update [12].

In recent years, data mining and machine learning have made a lot of progress in the fields of exploiting vulnerabilities. An earlier study was based on code similarity for C programs, Yamaguchi et al. [13] embedded Application Program Interface(API) usage patterns in a vector space and applied Principal Component Analysis(PCA) to detect similar vulnerabilities in the same type structure as the provided original sample. Soon later, Yamaguchi et al. [14] put forward the concept of Code Property Graphs (CPGs) which is a joint representation of a program’s syntax, control flow, and data flow. Based on this theory, Backs et al. [15] transformed PHP code into CPGs and identified vulnerabilities via graph traversals. Russell et al. [16] created a custom C/C++ lexer. Then they combined feature-extraction approaches of random forest(RF) [17] and Convolutional Neural Network (CNN) [18] to develop a function-level vulnerability detection model. VulDeePecker [19] is a vulnerability detection system based on deep learning. It focuses on library/API function calls and uses custom code gadgets to transform C/C++ codes into vectors. Through experiments, the authors found Bidirectional LSTM (BiLSTM) network [20] achieved the best effect. NAVEX [21] checks the sinks, sanitizations, traversal types and attack strings according to the prescribed dictionaries. Then, it obtains the data stream by traversing the generated CPGs. Especially, NAVEX combines dynamic execution methods to generate concrete exploits. NAVEX can detect SQL injection, XSS, Execution After Redirect(EAR) [22] and command injection vulnerabilities.

Most of the above tools and models are limited to testing a few vulnerabilities, and some of them use an auxiliary sink list to find possible vulnerability functions. The sink list mechanism needs experts to find the feature functions triggered by the vulnerability in time when a new vulnerability occurs. It is the same problem as traditional tools. Most scholars are inclined to generate complex graphs. However, the cost of building a graph structure is very high, and most generator is based on third-party tools. For solving those problems, we designed TAP which is based on the token mechanism and obtains multiclass vulnerabilities knowledge automatically only by inputting vulnerable and safe samples.

## Methods

**Fig 1:**
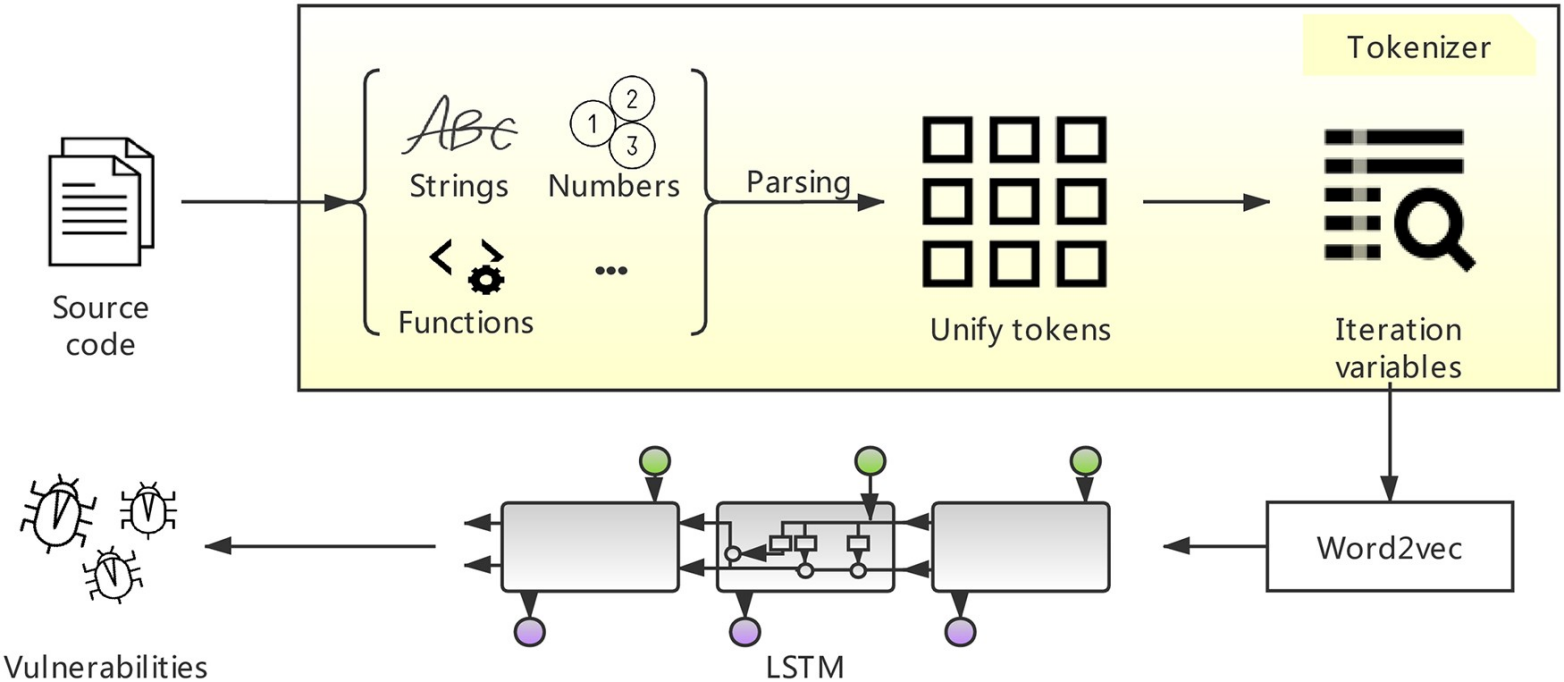
Overview of TAP.

We put forward some tokenized methods to deal with PHP code and data flow. The flow chart of TAP is shown in the Fig 1. According to features of PHP, our custom tokenizer particularly parses strings, numbers and functions based on the PHP original token mechanism. After unifying tokens, the tokenizer iterates variables to achieve data flow analysis, and it will only preserve lines of code with functions. Then we use word2vec model to obtain vectors and use the LSTM network to train TAP. Finally, TAP can recognize more than 7 categories of vulnerabilities.

**Fig 2:**
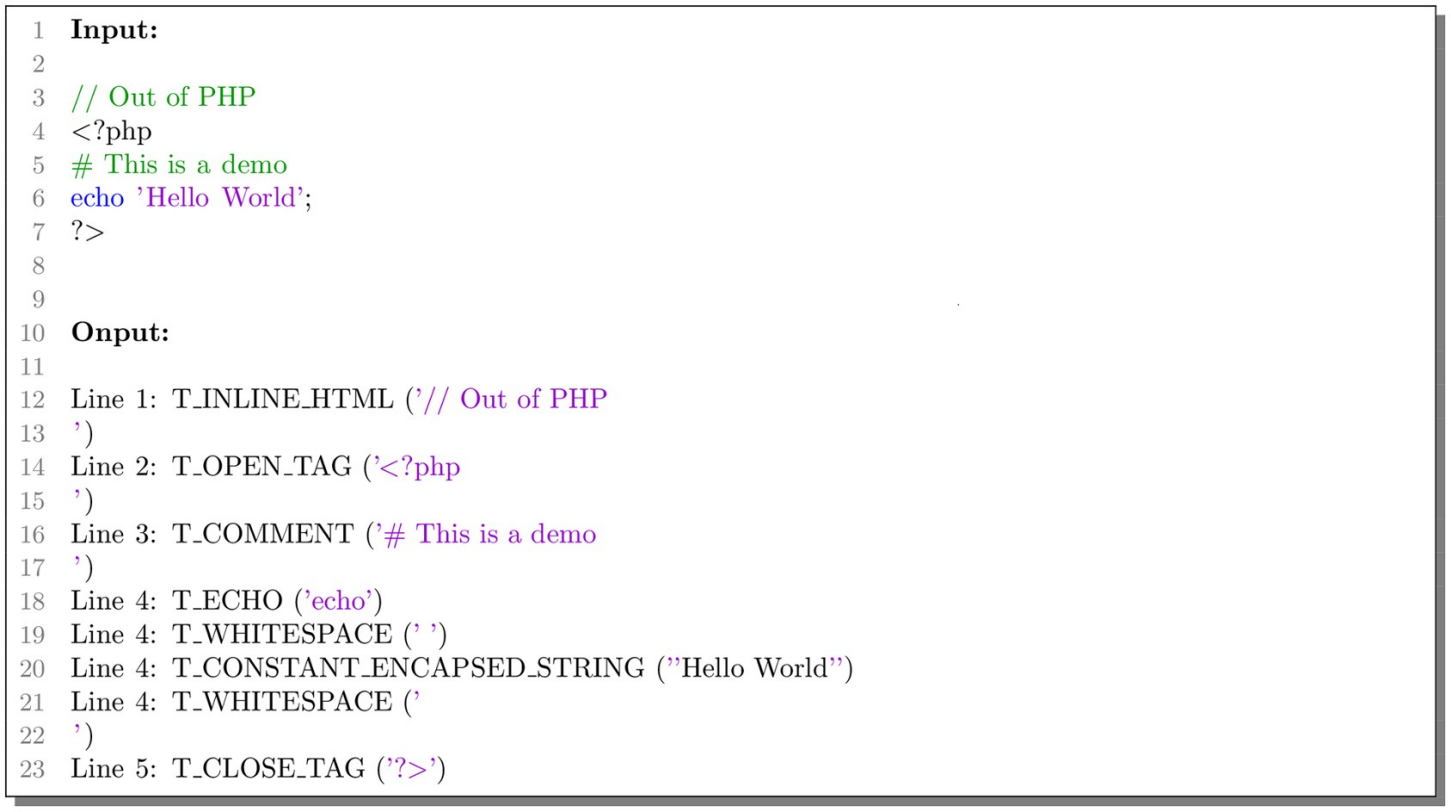
Example of PHP inbuilt function.

The PHP inbuilt function *token_get_all()* can parse code into PHP tokens by Zend engine [23]. Each PHP keywords and symbols will be translated into a word which begins with a capital letter T. As shown in the Fig 2, function *token_get_all()* can recognize HTML code, comments and composite symbols, and it can also locate the line where the code appears. But this function will parse any strings into the same token *T_CONSTANT_ENCAPSED_STRING*, and it is adverse to extract information from strings because of losses of any string content details. The same is true of numbers.

Besides, function *token_get_all()* only recognizes PHP keywords and few of functions. Other functions and identifiers like class names are all parsed into token *T_STRING* [24], and it will be very difficult for the deep learning model to recognize PHP code. Thus it is necessary to optimize standard PHP tokenizer.

### Parsing strings

According to our previous knowledge about vulnerabilities, we deem that the boundary symbols of strings are more likely to associate with SQL injection and other vulnerabilities than the contents of whole strings. So the custom tokenizer scans every string to extract boundary symbols. For example, a constant string SELECT * FROM A where A.B =’ will be only parsed into *T_CONSTANT_ENCAPSED_STRING* by inbuilt function *token_get_all()*, but the tokenizer of TAP will parse it into *T_CONSTANT_ENCAPSED_STRING*’ for emphasizing the single quote.

Besides, there is a PHP specific case that variables will be parsed when they are between double quotation marks. So the boundary symbol does not only mean the first and last characters of strings, but also means the characters adjacent to variables between double quotation marks.

**Fig 3:**
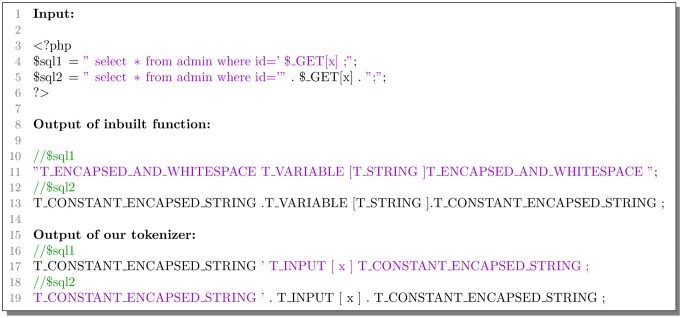
Comparison of parsing different boundary situations.

As shown in Fig 3, *$sql1* and *$sql2* are the different expressions of the same SQL query sentence. PHP inbuilt function *token_get_all()* parsed them into different token sequences. In particular, *$sql1* has extra delimiter quotation marks for strings which are meaningless. This inconsistency would cause the model to handle codes incorrectly.

Our tokenizer can perfectly recognize different boundary situations and the special characters of the boundary. Besides, our tokenizer unifies tokens of various forms for facilitating identification.

### Parsing numbers

Integer type numbers are usually used in config options, and lots of misconfigure issues just because of a wrong integer. But if all integers are parsed into the same token *T_LNUMBER*, our model will have no idea what the real config options are and cannot locate this type of vulnerability. For solving this problem, we decide to keep the original value of integer type number, which means the tokenizer of TAP will not parse integers and integer type variables into tokens.

### Parsing functions

Function *token_get_all()* cannot obtain the information of function names, because all function names are parsed into token *T_STRING*. We use function *function_exists()* to judge whether they are defined functions or not. Next, our tokenizer will keep the function name when it is a defined function or its token is *T_STRING*. Besides, several functions of old versions are abolished by upgrading PHP 7, so we collect and remain abandoned functions which are not recognized by PHP 7’s function *function_exists()* for downward compatibility.

### Unifying tokens

The names of functions are different from each other, in fact, some functions implement similar or even the same purpose. For preventing the deep learning model from getting entangled in the different names of the same functions, we maintain a list that is used to unify the similar functions’ tokens. The whole list is shown in Table 1.

**Table 1:** List of uniform tokens.

Uniform tokens	Token names or function names
T_IGNORE	T_DOC_COMMENT, T_COMMENT, T_INLINE_HTML, T_WHITESPACE, T_OPEN_TAG, T_CLOSE_TAG
T_ASSIGNMENT	T_AND_EQUAL, T_CONCAT_EQUAL, T_DIV_EQUAL, T_MINUS_EQUAL, T_MOD_EQUAL, T_MUL_EQUAL, T_OR_EQUAL, T_PLUS_EQUAL, T_SL_EQUAL, T_SR_EQUAL, T_XOR_EQUAL
T_COMPARISON	T_IS_EQUAL, T_IS_GREATER_OR_EQUAL, T_IS_IDENTICAL, T_IS_NOT_EQUAL, T_IS_NOT_IDENTICAL, T_IS_SMALLER_OR_EQUAL
T_INCLUDES	T_INCLUDE, T_INCLUDE_ONCE, T_REQUIRE, T_REQUIRE_ONCE
T_ECHO	T_PRINT, T_ECHO, T_EXIT, T_OPEN_TAG_WITH_ECHO, print_r, printf, vprintf, trigger_error, user_error, odbc_result_all, ifx_htmltbl_result
T_INPUT	$_GET, $_POST, $_COOKIE, $_REQUEST, $_FILES, $_SERVER, $HTTP_GET_VARS, $HTTP_POST_VARS, $HTTP_COOKIE_VARS, $HTTP_REQUEST_VARS, $HTTP_POST_FILES, $HTTP_SERVER_VARS, $HTTP_RAW_POST_DATA, $argc, $argv, get_headers, getallheaders, get_browser, import_request_variables
T_PREG	preg_filter, preg_grep, preg_last_error, preg_match_all, preg_match, preg_quote, eregi, preg_replace_callback, preg_replace, ereg_replace, ereg, eregi_replace,
T_EXEC	backticks, exec, expect_popen, passthru, pcntl_exec, popen, eval, proc_open, shell_exec, system,
T_SQL	dba_insert, dba_fetch, dba_delete, dbx_query, odbc_do, odbc_exec, odbc_execute, db2_exec, db2_execute, fbsql_db_query, fbsql_query, ibase_query, ibase_execute, ifx_query, ifx_do, ingres_query, ingres_execute, ingres_unbuffered_query, msql_db_query, msql_query, mssql_query, sybase_unbuffered_query, mssql_execute, mysql_db_query, mysql_query, mysql_unbuffered_query, mysqli_stmt_execute, mysqli_query, mysqli_real_query, mysqli_master_query, oci_execute, ociexecute, ovrimos_exec, ovrimos_execute, ora_do, ora_exec, pg_query, pg_send_query, pg_send_query_params, pg_send_prepare, pg_prepare, sqlite_open, sqlite_popen, sqlite_array_query, arrayQuery, singleQuery, sqlite_query, sqlite_exec, sqlite_single_query, sqlite_unbuffered_query, sybase_query,

For example, in the fifth line of the Table 1, functions *include()*, *include_once()*, *require()* and *require_once()* are parsed into 4 different token names by PHP inbuilt function. However, they all implement the same purpose which is importing another local file, so our tokenizer parses them into the same token *T_INCLUDES*. We can not recognize all functions or variables through token names parsed by inbuilt function. Thus we have to combine token names with function names to unify tokens.

As a special case, tokens which are unprofitable to analyze will be eliminated, the second line of Table 1 is the ignored tokens list.

### Data flow analysis

**Fig 4:**
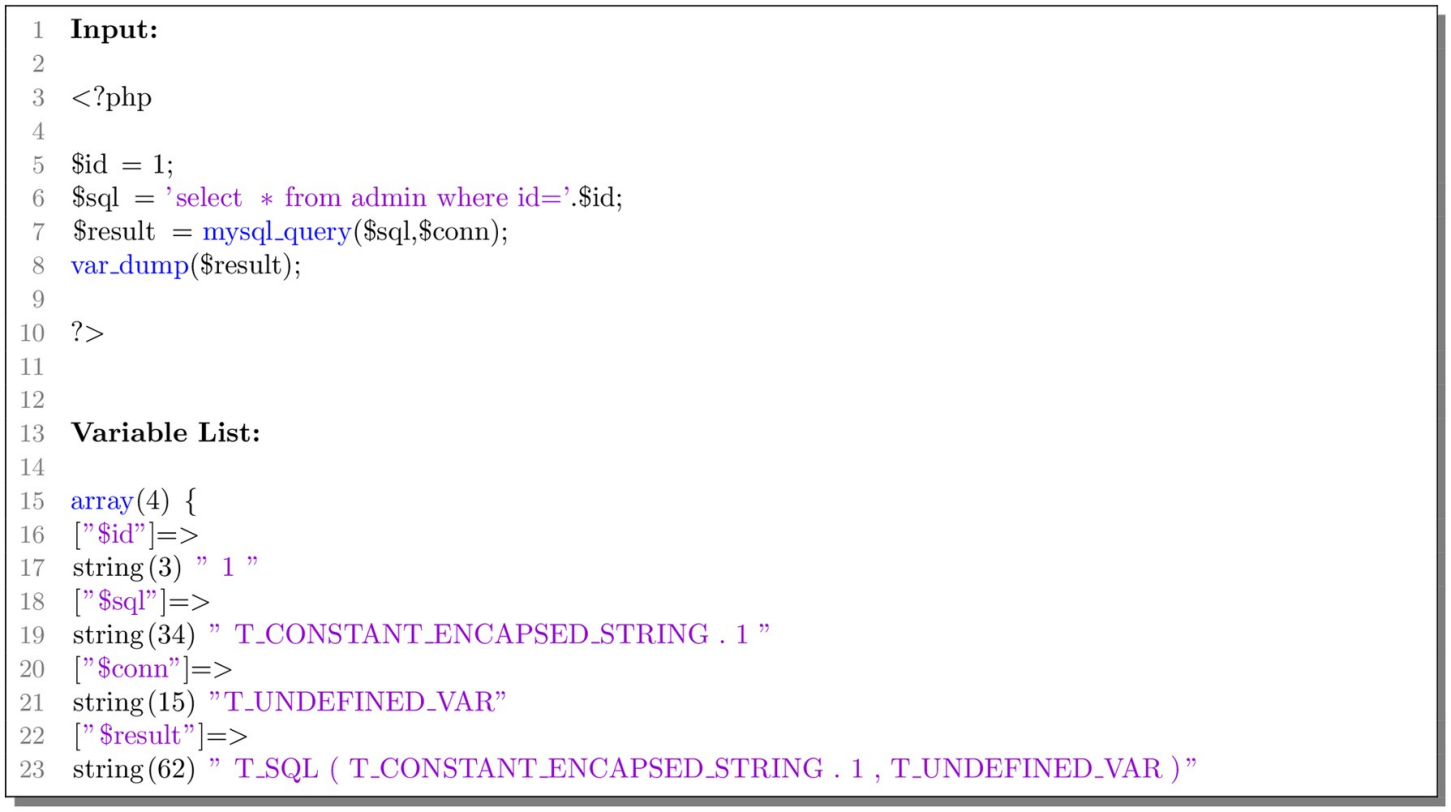
Example of variable iterations.

It is a nodus that tracking the variable change in static code analysis. The custom tokenizer records and iterates the changes of variables when a line of code is an assignment statement. It sounds like symbolic execution [17], but our method does not execute the PHP code and only records operations on variables. Our method not only reduces the difficulty of implementation and time cost but also achieves the work of data flow analysis. As shown in Fig 4, the variable *$result* only records that function *mysql_query()* processed *$sql* and *$con*, and it does not obtain the execution result of this line of codes.

According to our understanding of PHP codes, we found that one line of vulnerable codes must include at least one function, other code statements such as assignment and definition will not be directly linked to the vulnerabilities.

Therefore, the tokenizer of TAP will only keep lines of codes with functions and substitute the iterative variable value for the corresponding variable name. These can conduce to the deep learning model dealing with parameter passing and discover the lines of codes directly related to vulnerabilities.

**Fig 5:**
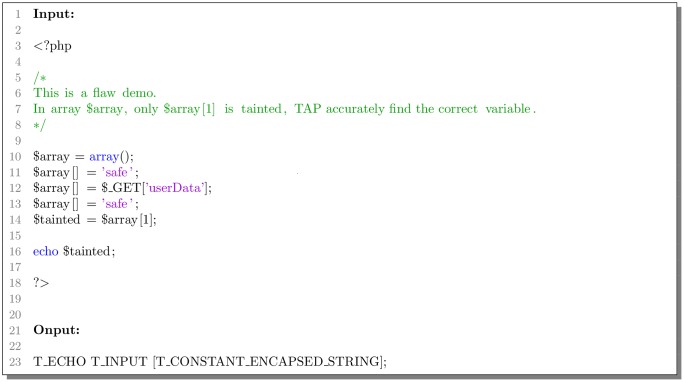
Example of the TAP’s custom tokenizer.

The final result of TAP’s custom tokenizer is shown in Fig 5. Our tokenizer only remains the last line of codes because it executes a function. Moreover, it can be found that the tokenizer accurately recognizes the parameter content of the echo function is the second item of the array variable.

### Algorithm model

#### Embedding

One-hot encoding is a primary embedding method that outputs a discrete sparse matrix. In essence, it is a bag of words model. One-hot encoding ignores the order of words and assumes that words are independent of each other. Word2vec [25] is a kind of word embeddings methods that can transform the corpus of text into a vector space by using a neural network. It can preserve the semantic and syntactic relationships.

We consider that program language is similar to natural language. The meaning of words influences each other, and the sequence of words should be treated with caution because a different position of a word may make the meaning of a sentence opposite. Besides, our tokenizer parses user-defined functions as tokens. Vectors will be too sparse and cause dimensional disasters after one-hot encoding. Thus we choose word2vec rather than one-hot encoding.

#### Basic model

CNN algorithm supposes that input and output are also independent, and elements are independent of each other. CNN is often used in image recognition field. Recurrent Neural Network(RNN) has an internal state to process input sequences, and it is suitable for sequencing data such as voice record, text data and so on. But there are the exploding and vanishing gradient problems in the RNN model.

LSTM network [26] is an improved RNN [27] for dealing with long-term dependencies. There are an input gate, an output gate and a forget gate in an LSTM network unit to solve the exploding and vanishing gradient problems in traditional RNN. It means the LSTM model can remember more history information and distant words than RNN. Thus we consider that the LSTM network is more suitable for our datasets, the experience also proves it.

## Experimental

### Dataset

#### SARD

Bertrand Stivalet and Elizabeth Fong [28] proposed a method to generate PHP test cases automatically. The generated PHP test cases were uploaded to the SARD, which is a subject of the National Institute of Standards and Technology(NIST) [29].

All 42212 files are consisting of 29258 safe samples and 12954 unsafe samples in provided datasets. Those vulnerable samples contain 12 categories of Common Weakness Enumeration(CWE) vulnerabilities, such as CWE-78: OS Command Injection, CWE-79 Cross-site Scripting and so on. It is necessary for deep learning model to be offered sufficient data, so we had to discard three subsets of less than 10 samples, which are CWE-209: Information Exposure Through an Error Message, CWE-311: Missing Encryption of Sensitive Data and CWE-327: Use of a Broken or Risky Cryptographic Algorithm.

The statistics of PHP test cases we used are shown in Table 2. In our experiment, the datasets are divided into the training dataset, validation dataset and test dataset with the ratio of 7:1:2.

**Table 2:** The statistics of datasets.

CWEs	Safe samples	Vulnerable samples	Totals
CWE-78: OS Command Injection	1872	624	2496
CWE-79: Cross-site Scripting	5728	4352	10080
CWE-89: SQL Injection	8640	912	9552
CWE-90: LDAP Injection	1728	2112	3840
CWE-91: XML Injection	4784	1264	6048
CWE-95: Eval Injection	1296	336	1632
CWE-98: PHP Remote File Inclusion	2592	672	3264
CWE-601: Open Redirect	2208	2592	4800
CWE-862: Missing Authorization	400	80	480
Totals	29248	12944	42192

#### SQLI-LABS

SQLI-LABS is a CWE-89 SQL injection training application for the security researcher, and it contains SQL injection vulnerability in more than ten different situations. We have sorted out 69 sample files related to vulnerability. Also, the datasets are divided into the training dataset, validation dataset and test dataset with the ratio of 7:1:2.

### Experiments

To evaluate TAP, we performed experiments by using a Ubuntu server with a 4-core 3.6 GHz Intel Core i7-7700 processor, a 6GB GeForce GTX 1060 Graphics Processing Unit(GPU) and 16GB memory.

First of all, we manually checked all the datasets and found many errors in the PHP test cases. As shown in the Table 3, we found 800 variable misused errors in 800 files and replaced the undefined constant “checked_data” with the right variable “$tainted” which is a tainted input according to context. We found 192 handwriting errors in 96 files, “tainted” was wrongly written as “tained” in those files. In 192 safe sample files, we found another 960 handwriting errors that the assignment symbol in the array was written as “= >” whose correct form is “=>”. Another problem is quotation marks misuse errors. There are the curly double open quote and curly double close quote which the PHP program cannot recognize in 4 files.

**Table 3:** Errors found by manul check.

Errors	Corrections	Safe samples (errors/files)	Vulnerable samples (errors/files)	Totals (errors/files)
checked_data	$tainted	448/448	352/352	800/800
tained	tainted	192/96	0/0	192/96
= >	= >	960/192	0/0	960/192
”	″	4/2	4/2	8/4
“	″	4/2	4/2	8/4

We trained two models to compare the binary classification models and the multiclass classification models, respectively.

#### Binary classification model

We compared TAP with other algorithms on CWE-89 samples of the SARD and SQLI-LABS vulnerability dataset to prove the superiority of TAP in the binary classification.

After parsing, the word2vec model was trained based on training data. We thought every word had meaning even if it appeared only once, so parameter min_count is set to 1. The maximum distance between the current word and the prediction word in a sequence is 20. Each word will be translated into 256 dimensions via our word2vec model. To take into account the effect of all vectors, the maximum input length is set to 835 which is a litter larger than the maximum length of sequences. Since the LSTM network must input a fixed-length sequence, the insufficient input tokens will be padded with zeros.

The number of safe samples is more than 9 times that of vulnerability samples. In fact, it is a very common situation in the real world, most of the codes are safe, and some type of vulnerabilities are rare. Imbalanced positive and negative samples will lead to a biased model, so the weight of each category should be adjusted. We increased the weight of categories with fewer samples and reduced the weight of categories with more samples. The weight is inversely proportional to the amount of data and all weights add up to 1.

**Fig 6:**
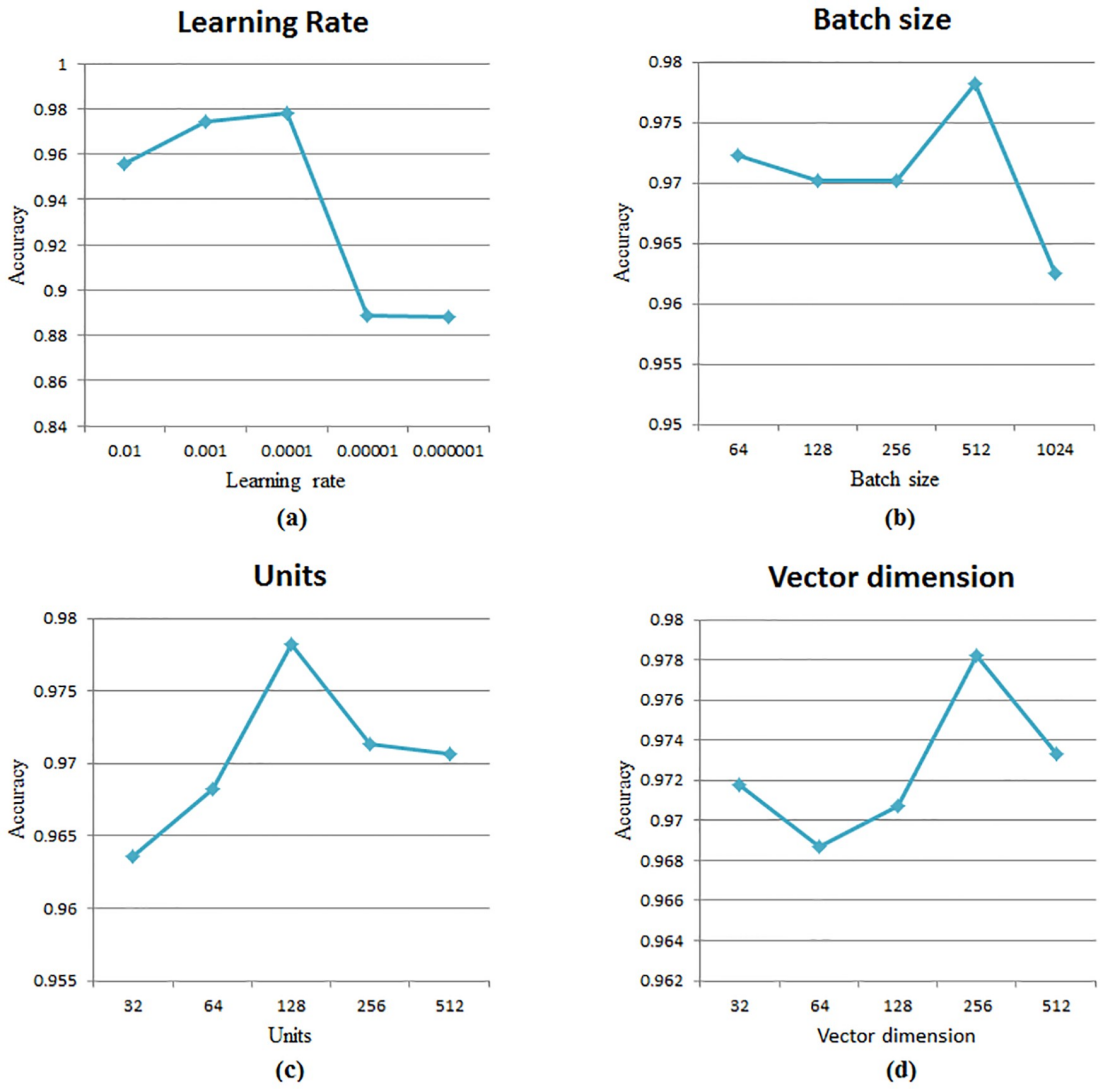
Control experiments of model parameters.

We added an LSTM network layer and a 50% dropout layer for preventing overfitting. The last layer is a 1 unit dense layer with “sigmoid” activation. We performed dozens of comparative experiments to evaluate the parameters and selected a few key parameters to make an intuitive graph as shown in the Fig 6. By comparing the results, we decided to adopt the following values of parameters. The dimension of vector is set to 256, the number of LSTM units is set to 128, the value of batch size is set to 512, the epochs are set to 60, the loss function is set to “mse”, the optimizer is “adam” and the number of learning rate is set to 0.0001.

#### Multiclass classfication model

TAP was compared with other tools on all categories of the SARD datasets to prove the ability of multiclass classifications.

After correcting errors, all the datasets are divided into 10 categories, including 9 categories of CWE vulnerabilities and a safe category comprises all safe samples. Next, we parsed raw codes into tokens by our custom tokenizer.

Other parameters are the same as the binary classifier model, except some config of neural network. The length of the longest input sequence is set to 350. Besides, this is a 10 categories classification problem, so the last output layer is a 10 units dense layer with “softmax” activation, and loss function is set to “categorical_crossentropy”.

## Evaluation

### Evaluation criteria

#### F1 score

The F1 score is an important metric of evaluating security models [30]. It also takes into account the accuracy and recall rate of the classification model.

First, we will introduce some basic concepts:

True Positive(TP) is the number of samples where the predicted and the real label are both positive.

False Positive(FP) is the number of samples where the predicted label is positive, but the real label is negative.

True Negative(TN) is the number of samples where the predicted and the real label are both negative.

False Negative(FN) is the number of samples where the predicted label is negative, but the real label is positive.

The formulas for precision and recall are as follows:

$$\begin{array}{c} \text{Precision}=\frac{TP}{TP+FP} \end{array}$$ (1)

$$\begin{array}{c} \text{Recall}=\frac{TP}{TP+FN} \end{array}$$ (2)

Thus, the complete F1 score formula are as follows:

$$\begin{array}{c} F1=2\cdot \frac{\text{precision}\cdot \text{recall}}{\text{precision}+\text{recall}} \end{array}$$ (3)

#### ROC

Receiver Operating Characteristic(ROC) [31] curve is usually used to evaluate the diagnostic ability of binary classifiers. The *x*-axis is the False Positive Rate(FPR) and the *y*-axis is the True Positive Rate(TPR). According to the predicted probability value and the real label of each test sample, different points predicted by the model can be obtained by adjusting the threshold. These points can be connected to a curve, which is called the ROC curve. The larger the AUC, the better the effect of the model.

$$\begin{array}{c} \text{TPR}=\frac{\text{TP}}{\text{TP}+\text{FN}} \end{array}$$ (4)

$$\begin{array}{c} \text{FPR}=\frac{\text{FP}}{\text{FP}+\text{TN}} \end{array}$$ (5)

#### Confusion matrix

Multiclass classification models usually use the confusion matrix to illustrate diagnostic ability. The confusion matrix is a unique table used to show the classification effect of models visually. The rows and columns of the table are the real categories and the categories predicted by the model, respectively. Assuming that *x*-axis is predicted category and *y*-axis is the real category, then the numbers in cells of row *i* and column *j* represent the number of samples that belongs to category *i* but are predicted to category *j*. Thus, the data on the diagonal from the top left to the bottom right shows that the number of correct classifications.

In general, specific numerical values are filled in the form, and sometimes the regularized results can also be filled in. The regularized number in the cell of row *i* and column *j* is the number in the cell of row *i* and column *j* divides by the sum of the numbers of cells in row *i*.

#### Kappa statistic

Kappa statistic is used for checking consistency and measuring classification accuracy. The calculation of the kappa coefficient is based on the confusion matrix.

$$\begin{array}{c} \kappa \equiv \frac{p_{o}-p_{e}}{1-p_{e}}=1-\frac{1-p_{o}}{1-p_{e}} \end{array}$$ (6)

*p*_*o*_ is the overall classification accuracy. *p*_*e*_ represents the probability that the prediction results are consistent with the monitoring results by accident. Assuming that *k* is the total number of categories and *N* is the total number of samples, *n*_*ij*_ indicates the number of categories *i* but predicted to be *j*.

$$\begin{array}{c} p_{o}=\frac{1}{N}\sum_{i=1}^{k}n_{ii} \end{array}$$ (7)

$$\begin{array}{c} p_{e}=\frac{1}{N^{2}}\sum_{i=1}^{k}\left(\sum_{j=1}^{k}n_{ij}\sum_{j=1}^{k}n_{ji}\right) \end{array}$$ (8)

Kappa value ranges from -1 to 1, but it is generally between 0 and 1 to evaluate the effect [32]. The detailed evaluation standard is shown in Table 4.

**Table 4:** Effect evaluation of Kappa statistic.

Kappa Statistic	<0.00	0.00-0.20	0.21-0.40	0.41-0.60	0.61-0.80	0.81-1.00
Strength of Agreement	Poor	Slight	Fair	Moderate	Substantial	Almost Perfect

#### Hamming distance

Hamming distance measures the degree of inconsistency between the predicted categories and the real categories of samples. If *y* is a test dataset, *y*_*j*_ is the real value of a given sample of *j*-th label, ${\hat{y}}_{j}$ is the predicted value, *n* is the number of labels, and 1(*x*) is the indicator function, then the Hamming distance is

$$\begin{array}{c} D_{Hamming}\left(\hat{y},y\right)=\frac{1}{n}\sum_{j=0}^{n-1}1\left({\hat{y}}_{j}\neq y_{j}\right) \end{array}$$ (9)

The distance is 0 when the prediction result is completely consistent with the true situation. When the prediction result is completely inconsistent with the true situation, the distance is 1; when the prediction result is partially correct, the distance is between 0 and 1. The smaller the value, the better.

### Results and discussion

#### Comparison of binary classification models

First, the custom tokenizer of TAP was compared with the inbuilt function of PHP on the CWE-89 dataset to prove the effectiveness of the custom tokenizer. The datasets includes the CWE-89 dataset of SARD and samples of SQLI-LABS. Since the length of the tokens parsed by the two models is different, the other parameters are the same except that the maximum input length.

**Fig 7:**
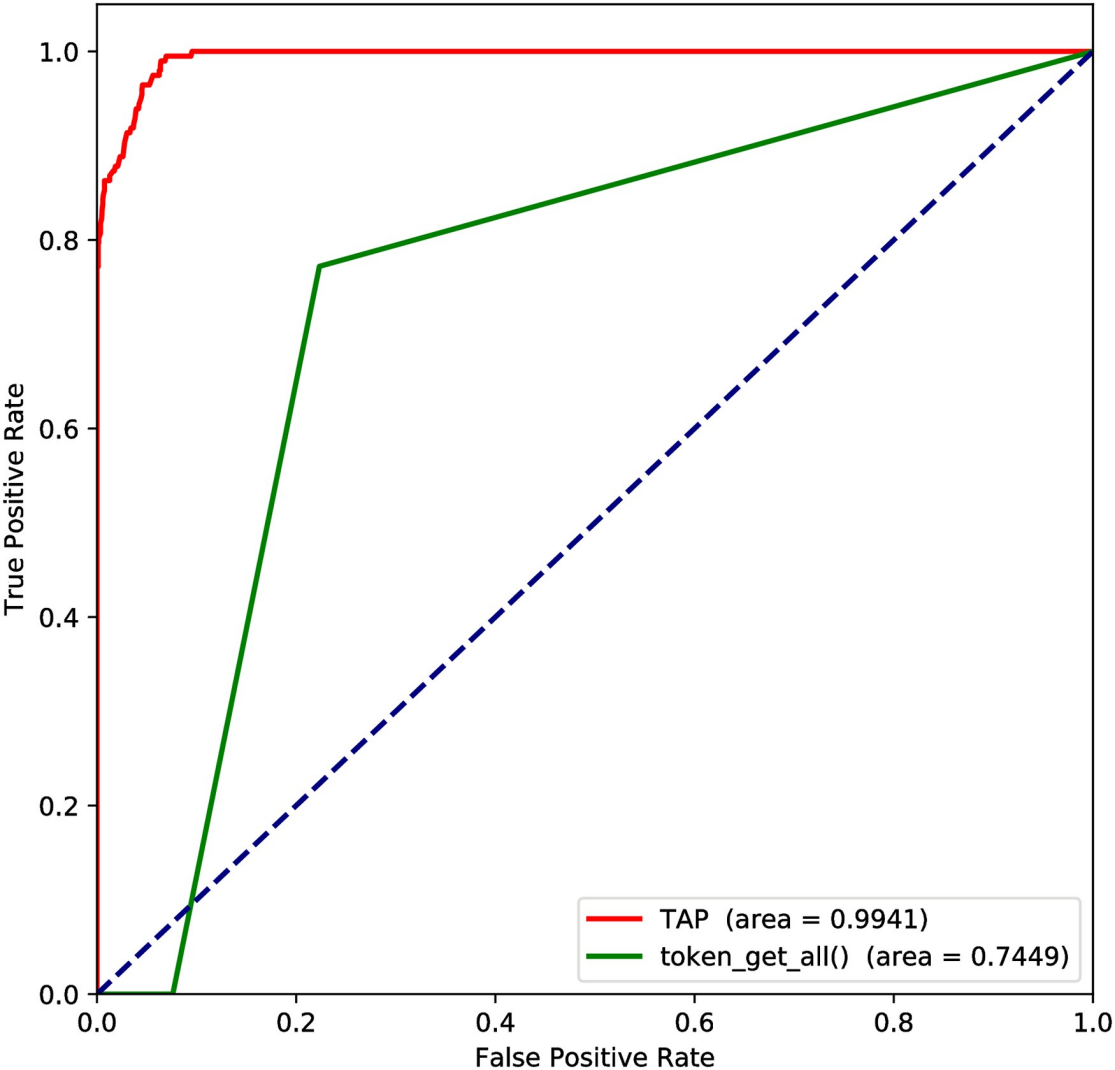
Comparison of TAP and token_get_all().

The AUC of builtin function *token_get_all()* is less than 0.5, so we reverse the result. The ROC curves of the two models are shown in the Fig 7. Besides, other evaluation criteria are listed in Table 5, we can find that the custom tokenizer of TAP is much better than function *token_get_all()* in multiple metrics. In the vulnerable sample, the precision of *token_get_all()* model is perfectly 1, but the recall is almost zero. Thus the high precision is little significance, the F1 score also proves this.

**Table 5:** Results comparison with TAP and token_get_all().

Method	Safe samples			Vulnerable samples			accuracy	AUC
	Precision	Recall	F1	Precision	Recall	F1		
TAP	**0.9773**	0.9988	**0.9880**	0.9874	**0.7970**	**0.8820**	**0.9782**	**0.9941**
token_get_all()	0.9047	**1.0**	0.9499	**1.0**	0.0761	0.1415	0.9055	0.7449

Next, we compared TAP with other models to prove the superiority of TAP. The models include BiLSTM, CNN, RNN, Gated Recurrent Unit(GRU), RIPS and WIRECAML [33]. WIRECAML is a static code analysis model based on machine learning and data-flow analysis. The author of WIRECAML compared WIRECAML with other machine learning algorithms such as random forest, logistic regression, naive Bayes and so on. WIRECAML performs best in all the machine learning algorithms, so we consider that WIRECAML represents one of the best static code analysis models based on machine learning. The hyper-parameters of TAP, BiLSTM, CNN, GRU and RNN are the same.

We modified WIRECAML [34] program to keep the datasets consistent and obtain the required intermediate variables. WIRECAML predicts vulnerabilities per line of codes and gives an overall result. In order to get the predicted probability value of a single file, we select the maximum probability value of the source code file as the probability of the whole file.

For evaluating RIPS [35], we ran RIPS on the test dataset and selected the detected SQL injection vulnerabilities as evaluation criteria without other possible vulnerabilities. Besides, RIPS does not judge vulnerability based on probability values, so we can not calculate the ROC and AUC of RIPS.

**Fig 8:**
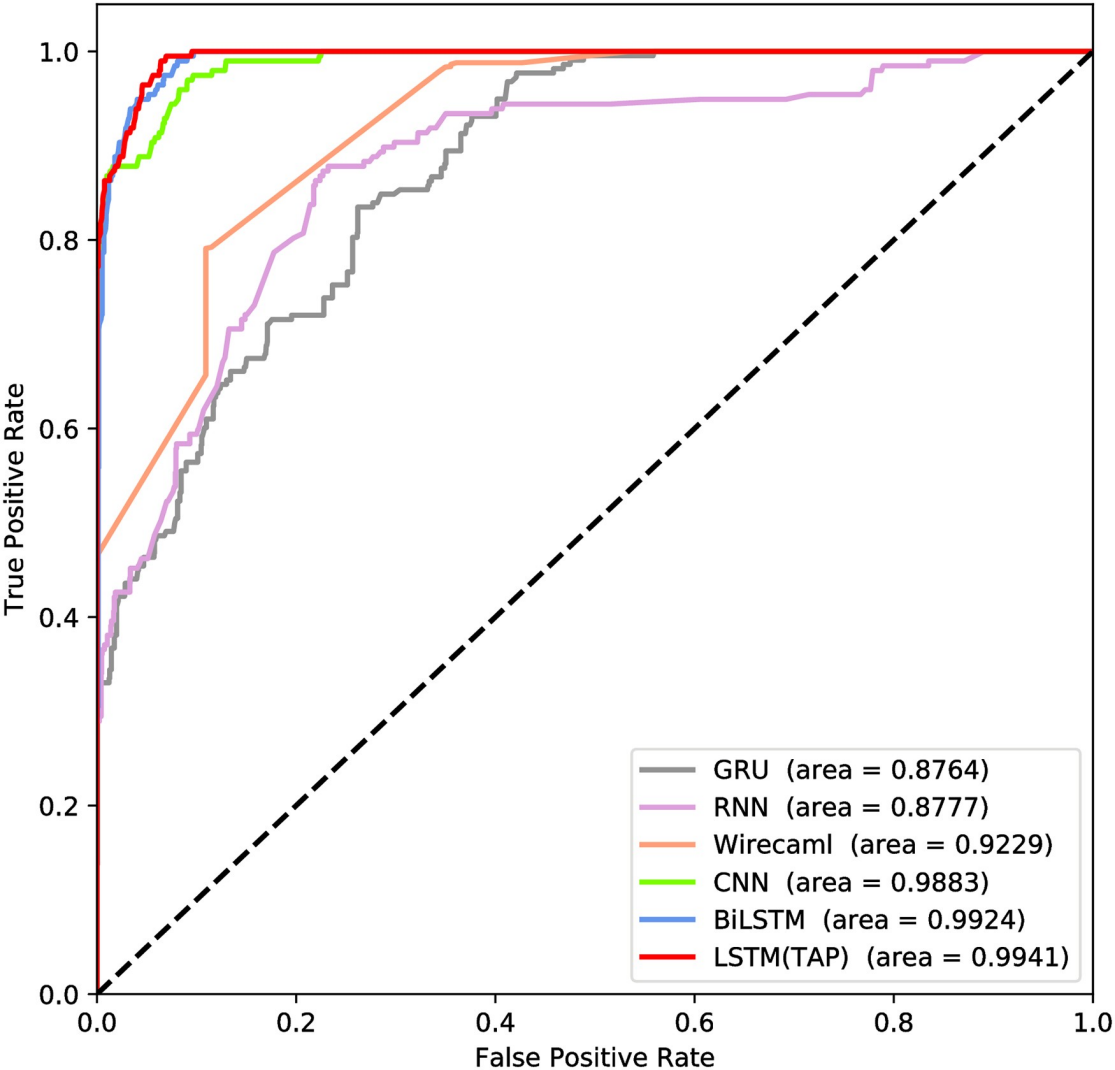
ROC comparison of binary classification model.

The ROCs are shown in Fig 8. As we can see, the GRU model appears the worst, TAP which used the LSTM network algorithm is the best.

Other evaluation criteria are listed in Table 6, the values in bold type are the best of the single standard. In the identification of security samples, the results of each model are good, but there is a big difference in the ability to identify vulnerable samples which we regard as more important. RIPS did not perform well on the test dataset, WIRECAML also had a low F1 score on vulnerable samples. GRU algorithm gets two perfect scores, but the F1 of vulnerable samples and AUC are too bad. The results of LSTM, BiLSTM and CNN are very similar. After comprehensive consideration, we consider that LSTM is the best.

**Table 6:** Evaluation criteria of binary classification model.

Algorithms	Safe samples			Vulnerable samples			accuracy	AUC
	Precision	Recall	F1	Precision	Recall	F1		
LSTM(TAP)	0.9773	0.9988	**0.9880**	0.9874	0.7970	**0.8820**	**0.9782**	**0.9941**
BiLSTM	0.9740	0.9902	0.9856	0.9061	**0.8325**	0.8677	0.9740	0.9924
CNN	**0.9789**	0.9942	0.9865	0.9412	0.8122	0.8719	0.9756	0.9883
GRU	0.9020	**1.0**	0.9485	**1.0**	0.1376	0.2419	0.9035	0.8764
RNN	0.9324	0.9896	0.9601	0.8022	0.3706	0.5069	0.9262	0.8777
WIRECAML	0.9631	0.9879	0.9753	0.8478	0.6393	0.7290	0.9548	0.9229
RIPS	0.9101	0.8027	0.8530	0.1496	0.3046	0.2007	0.7517	-

#### Comparison of multiclass classification models

In order to prove TAP’s ability to multiclass classification problems, we designed experiments on TAP and RIPS. We used RIPS to detect the 10 types of data subsets separately, and then manually counted and analyzed the results. The datasets are extremely imbalanced, the confusion matrix without normalization does not look intuitive and is not easy to evaluate the classification effect, so we use the normalized confusion matrix.

**Fig 9:**
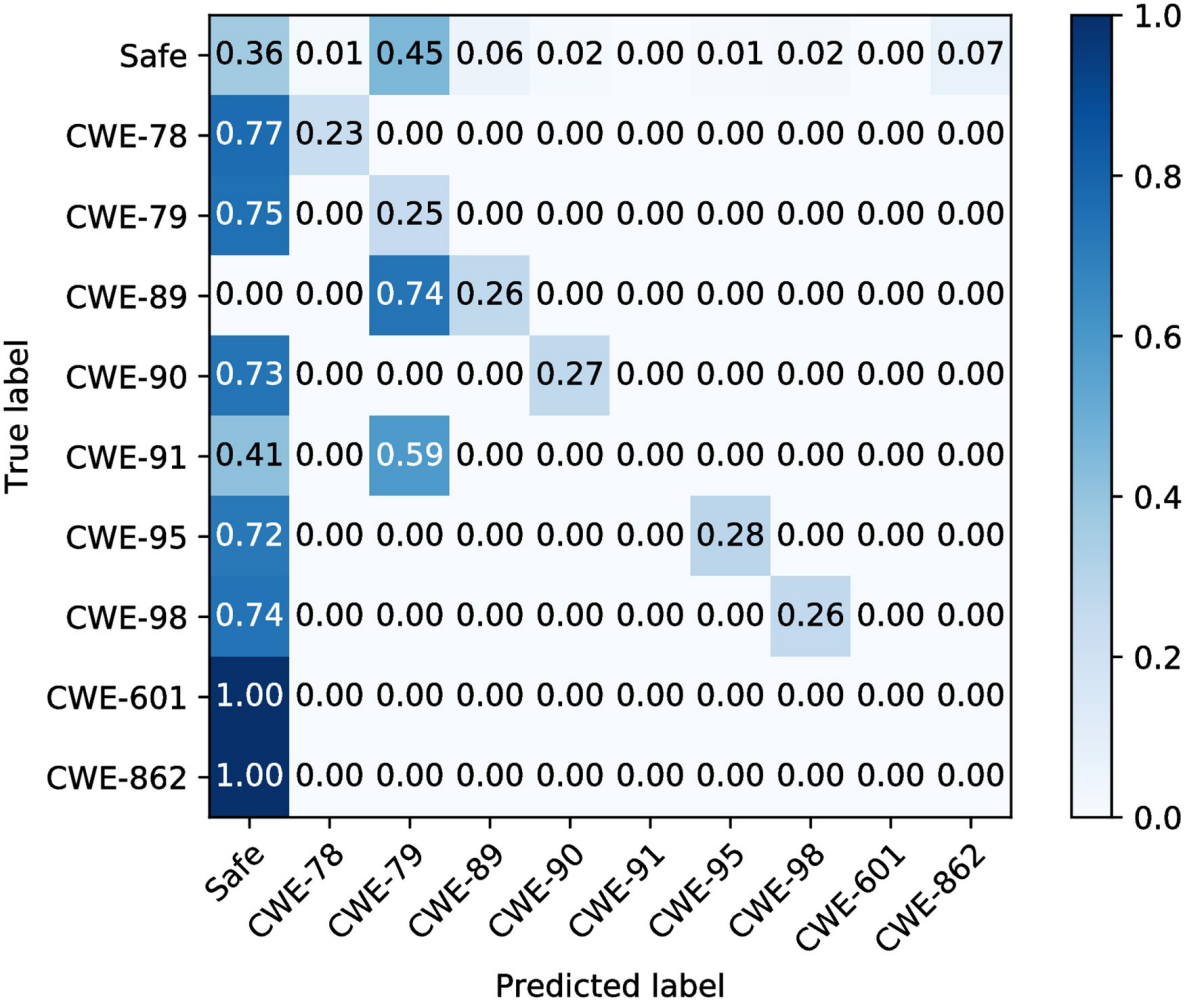
Normalized confusion matrix of RIPS.

**Fig 10:**
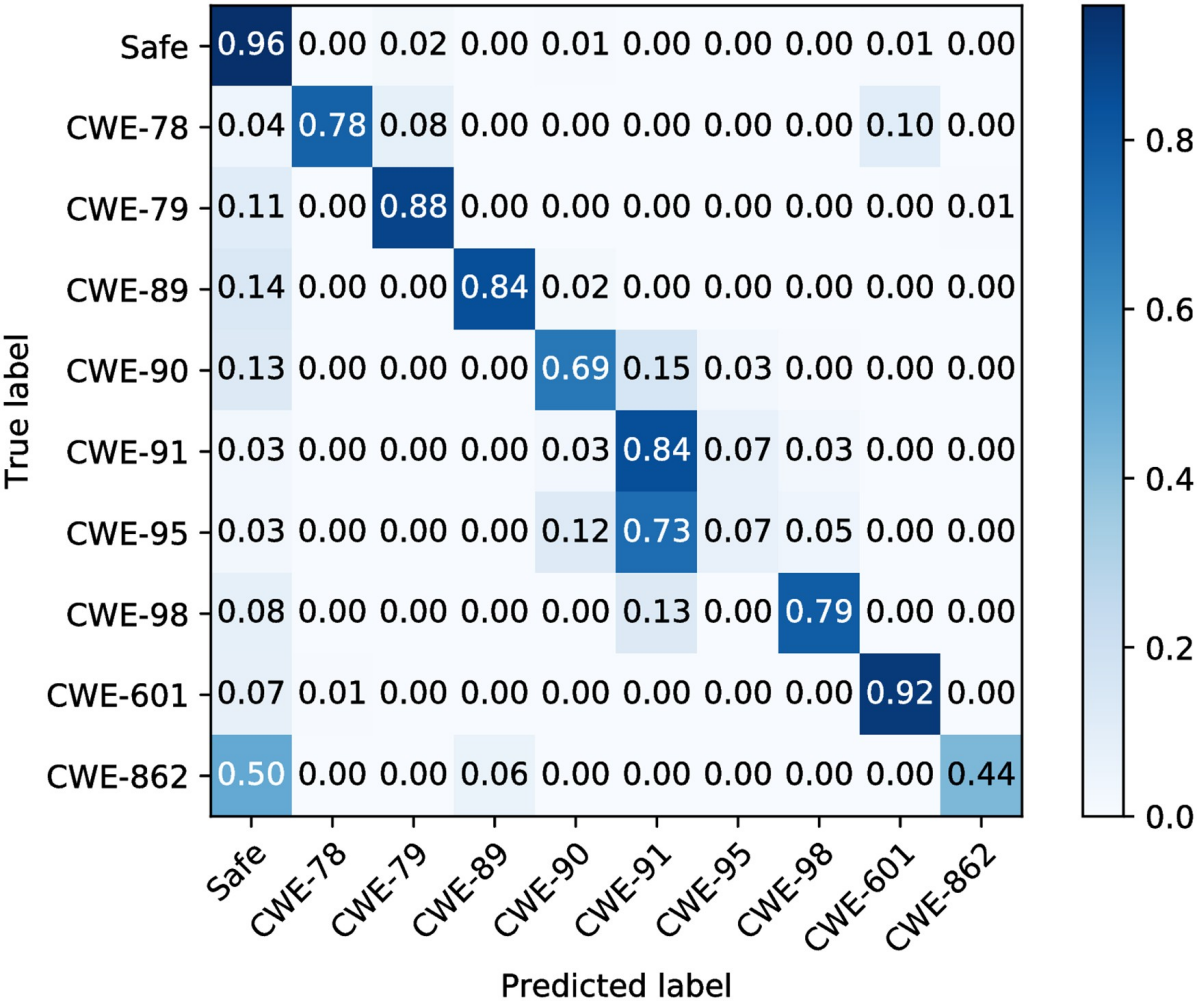
Normalized confusion matrix of TAP.

The classification results of RIPS and TAP are shown in Figs 9 and 10. RIPS mislabels some samples as vulnerabilities that are not included in the test dataset, and it also mislabels a single sample into multiclass vulnerabilities. In order to ensure the total number is correspond, we adjusted the number of mislabeled samples to the number which was obtained by subtracting the number of correctly identified samples from the total number of samples. For the samples with the safe label, we classify these mislabeled undefined vulnerabilities samples into CWE-862, and for the samples with the vulnerable label, we classify these mislabeled undefined vulnerabilities samples into safe. Because the built-in rules do not contain CWE-601 and CWE-862, RIPS mislabels all these two types of vulnerabilities. On the safe dataset, more than half of the samples are judged as vulnerable. On vulnerable dataset, most of them are judged as safe. It is obviously that RIPS cannot complete the multiclass classification task well on the test dataset with a very high misjudgment rate.

Overall, the multiclass classification ability of TAP is well. However, lots of samples of CWE-862 are mislabeled, and even almost all CWE-95 samples are mislabeled. After analysis, we think the most likely reason is that the numbers of these two categories of datasets are too few compared to other categories. Another possible reason is that the training datasets of CWE-91 and CWE-95 are similar in content or form after parsing.

Kappa and hamming distance of two models are shown in Table 7. According to Table 4, the Kappa of TAP indicates almost perfect, and the hamming distance is small enough. On the contrary, RIPS perform poorly both on Kappa and hamming distance.

**Table 7:** Evaluation criteria of multiclass classification model.

	Kappa	Hamming Distance
TAP	0.8319	0.0840
RIPS	-0.0985	0.6965

## Conclusion

This paper presents a static PHP source code analysis model named TAP, which based on the deep learning algorithm. It can help security researchers find vulnerabilities quickly. There is a custom tokenizer in TAP based on PHP inbuilt token mechanism. The tokenizer improves the parsing ability, completes parameter iteration and analyzes data flow. Experiments show that our optimization is indeed effective and the LSTM-based model TAP is superior to CNN, RNN, GRU, BiLSTM, WIRECAML and RIPS in vulnerable binary classification and multiclass classification.

For future work, we will support for processing object and more types of vulnerabilities. Besides, we want to extend TAP to the complex real-world environment.
